# Diabetes, Vascular Aging and Stroke: Old Dogs, New Tricks?

**DOI:** 10.3390/jcm10194620

**Published:** 2021-10-08

**Authors:** Theano Penlioglou, Anca Pantea Stoian, Nikolaos Papanas

**Affiliations:** 1 Diabetes Centre, Second Department of Internal Medicine, Democritus University of Thrace, 68132 Alexandroupolis, Greece; theanopen@gmail.com; 2 Diabetes, Nutrition and Metabolic Diseases Department, “Carol Davila” University of Medicine, 020021 Bucharest, Romania; ancastoian@yahoo.com

**Keywords:** diabetes mellitus, stroke, macrovascular complications, diabetes stroke

## Abstract

Background: Stroke remains a leading cause of death and disability throughout the world. It is well established that Diabetes Mellitus (DM) is a risk factor for stroke, while other risk factors include dyslipidaemia and hypertension. Given that the global prevalence of diabetes steadily increases, the need for adequate glycaemic control and prevention of DM-related cardiovascular events remains a challenge for the medical community. Therefore, a re-examination of the latest data related to this issue is of particular importance. Objective: This review aims to summarise the latest data on the relationship between DM and stroke, including epidemiology, risk factors, pathogenesis, prevention and biomarkers. Methods: For this purpose, comprehensive research was performed on the platforms PubMed, Google Scholar and EMBASE with a combination of the following keywords: diabetes mellitus, stroke, macrovascular complications, diabetic stroke, cardiovascular disease. Conclusions: Much progress has been made in stroke in people with DM in terms of prevention and early diagnosis. In the field of prevention, the adaptation of the daily habits and the regulation of co-morbidity of individuals play a particularly important role. Simultaneously, the most significant revolution has been brought by the relatively new treatment options that offer protection to the cardiovascular system. Moreover, many prognostic and diagnostic biomarkers have been identified, paving the way for early and accurate diagnoses. However, to date, there are crucial points that remain controversial and need further clarification.

## 1. Introduction

Today it is estimated that 1 in 11 adults live with diabetes mellitus (DM) [1]. The prevalence of DM is constantly increasing [1]. This holds true for both type 2 DM (T2DM) and type 1 DM (T1DM) [1]. Impaired glucose homeostasis and long-term exposure to hyperglycaemia increase the risk of developing microvascular (diabetic neuropathy, diabetic nephropathy and diabetic retinopathy) and macrovascular complications (myocardial infarction (MI), peripheral arterial disease and stroke) [2,3,4]. DM subjects exhibit a significantly higher risk of cardiovascular disease (CVD) compared with the general population [5]. Moreover, CVD, including stroke, is the leading cause of mortality [6,7]. In addition to their impact on the health and quality of life of DM subjects, macrovascular complications also have a negative impact on public health due to the high financial burden on health systems [7,8].

It is well established that DM represents an independent risk factor for stroke. In practice, DM subjects usually have other risk factors as well, notably hypertension, obesity and dyslipidaemia, which further increase the risk of stroke [8]. Due to its high mortality and disability rates, stroke in DM has received attention in terms of prevention and new biomarkers. Nevertheless, many issues still need further clarification.

Therefore, this review aimed to summarise the latest data on the relationship between DM and stroke, including pathogenesis, treatment and prevention.

## 2. Epidemiology and Risk Factors

DM is one of the most important vascular factors, affecting small and large blood vessels and causing various complications, which have been linked with stroke. It is an independent high-risk factor: subjects with DM have with a twofold risk of stroke [5,9,10]. Moreover, strokes in DM exhibit increased mortality. Indeed, in patients with DM, the relative death risk was increased by 1.8 (95% confidence interval (CI), 1.04 to 3.19) [11,12,13]. Interestingly, prediabetes is also linked with an increased risk of stroke [14]. Moreover, often DM is diagnosed after the occurrence of stroke, since many stroke patients have an undiagnosed DM or prediabetes [15].

According to a review of Asian data on stroke epidemiology [16], DM is the second most important factor after hypertension. Among DM subjects, the risk of stroke increases threefold [16]. In combination with other risk factors, the occurrence of stroke increases exponentially, compared with non-DM subjects sharing these risk factors [16]. Moreover, a prospective observational study [17] has shown that in T2DM subjects, stroke was commoner than MI. In this context, longer DM duration further increases the risk of stroke [18,19].

Several studies suggest that the incidence of stroke is similar between T1DM and T2DM, if not quite higher in T1DM [20,21]. Furthermore, T1DM is associated with a higher risk of premature stroke (10–15 years earlier) compared with non-DM individuals [22]. It is worth noting that this risk is even higher in women [23], especially in T1DM [23]. Very interesting and noteworthy is the fact that women with type 1 diabetes have a relative risk of all-cause death of 40% and double the excess risk of fatal and non-fatal vascular events compared to men with type 1 diabetes, according to data obtained in a meta-analysis that included 26 studies and 214,114 individuals [24]. On the other hand, data from the Malaysian National Stroke Registry, which included 4622 individuals with type 2 DM, showed no differences between genders regarding stroke recurrence with a previous history of stroke [25].

More intriguing are the Ontario healthcare database results, which included around 25,500 diabetic patients with index-ischemic stroke. The results have shown that women with DM have higher unadjusted mortality compared to men. However, female sex is not an independent risk factor. In addition, the incidence was higher in women than in men (95% confidence interval (CI), 13.73–14.44) versus 11.89 (11.60–12.19), and the recurrent stroke incidence was similar by gender, but men were more likely to be readmitted for MI (1.99 per 100 person-years (1.89–2.10) versus 1.58 (1.49–1.68) among females), and women had a lower risk for readmission for all-cause of CV events. Women with diabetes, compared to men, had higher unadjusted mortality and risk of readmission for any cause or death following an incident stroke but a lower risk of readmission for MI [26].

The role of body-mass index (BMI) is somewhat controversial. A prospective study in the Chinese population has shown that newly diagnosed patients with T2DM had a higher risk of ischaemic stroke, while those with a normal BMI also had a higher risk [27]. Li et al. [28] also found that each 1 kg/m^2^ increase in BMI was conversely associated with risk of ischaemic stroke in T2DM, while another group reported that a 5 kg/m^2^ increase in BMI increased the risk of stroke [29]. Other works have also shown increased CVD mortality in obesity [27,30,31].

The term “obesity paradox” refers to data that show that in people with chronic conditions, such as acute and chronic heart failures and coronary artery disease, increased BMI may play a protective role against mortality [32,33]. Various mechanisms have been proposed to explain the phenomenon, such as the reduction in NT-proBNP, whose high levels have been linked to high mortality in acute coronary disease, as well as the secretion of protective cytokines by the adipose tissue [34,35]. Regarding stroke, there are studies suggesting that increased BMI results in lower mortality rates [36,37]. In addition, other studies claim that it may reduce all-cause mortality in people with T2DM [38]. However, the presented data are controversial. On the one hand, BMI is an indicator that lacks reliability, as it cannot provide important data, such as the distribution of adipose tissue of the participants [36,39]. In addition, regarding DM, in several studies multiple aggravating factors, such as smoking, cause potential bias as they increase mortality [38].

Similarly, a Mongolian cohort study showed that DM combined with central obesity significantly increased the risk of stroke [40]. Subjects with both central obesity and DM exhibited the highest risk (73%) [40]. However, there is also a paradox: mortality is lower in overweight/obese subjects than in those whose BMI is normal or underweight [12,41].

In DM, hypertension is frequent and further increases the risk of both ischaemic and haemorrhagic stroke [42,43]. Other significant risk factors in DM include dyslipidaemia, previous MI and heart failure [44].

A Swedish study included all the patients who had undergone coronary artery bypass grafting for 12 years [45]. Both DM types were associated with an increased risk of stroke [45]. Impressively, mortality was significantly higher in T1DM and only slightly increased in T2DM [45]. Specifically, the all-cause mortality rate was 22% for T1DM and 21% for T2DM [45]. Moreover, in T1DM, inadequate glycaemic control and female sex further increased the risk of stroke [46,47]. The adverse influence of female sex contrasts with the reduced risk in non-DM women [48].

Smoking per se increases the risk of stroke, including in DM [49,50,51]. The results of a study in which 38,887 patients with T2DM participated, showed that smoke and T2DM are separately independent risks factors for stroke (OR 2.00, 95% CI 1.56 to 2.56; OR 1.65, 95% CI 1.36 to 2.00). In addition, the combination of those two factors increased the incidence of stroke (OR 3.45, 95% CI 2.30 to 5.16, *p* < 0.001). [49]. Smoking discontinuation may reduce [50] this risk or not [51].

There has been discussion about whether genetic background may increase the risk of stroke in DM, but the evidence is not conclusive [52,53]. Obviously, we need more knowledge on the interplay between DM and genetic predisposition.

Of practical relevance, meticulous care offered to DM subjects is important. A large prospective study found that intensified healthcare vs. standard care reduced the risk of stroke [54]. This therapeutic challenge awaits confirmation and broader implementation.

Finally, microvascular diabetic complications may play a role, as well. In a large Australian population study, diabetic retinopathy was associated with an increased risk of stroke, independently of previous major cardiovascular events [55]. These data could be explained by the hypothesis of “common soil”, since the two complications share the following common risk factors: hypertension, dyslipidaemia and hyperglycaemia [55]. According to Wang et al., among 751 diabetic patients who experienced stroke, the incidence of stroke rate was remarkably higher for patients with proteinuria. More specifically, it was 11.12 events per 1000 person-years for those with remittent proteinuria, 11.04 for those with incident proteinuria and 16.37 for those with persistent proteinuria, while for those without proteinuria it was mentioned to be 5.55 [56]. Therefore, remittent, persistent or incident proteinuria may also increase the risk of stroke [56]. Interestingly, this correlation seems to be stronger in prediabetes [56]. We now need to ascertain how retinopathy and proteinuria might be used as risk markers of the risk of stroke incidence in DM [55,56].

## 3. Clinical Manifestation

Both ischaemic and haemorrhagic stroke may occur in DM, but the latter is less frequent [57]. The Emerging Risk Factors Collaboration suggested that the adjusted hazard ratios (HRs) in DM were 2.27 (95% Confidence Interval (CI): 1.95–2.65) for ischaemic stroke, 1.56 (95% CI: 1.19–2.05) for haemorrhagic stroke and 1.84 (95% CI: 1.59–2.13) for unclassified stroke [57].

An interesting study has looked at patency in the circle of Willis arteries as well as posterior vs. anterior circulation [58]. In DM, the patency of the circle of Willis was commoner [58]. Moreover, DM was associated with commoner posterior circulation brain infarction and brain stem infarction [58].

One of the most common risk factors for ischemic stroke is intracranial stenosis, which is more prevalent in African American, Asian and Hispanic populations. DM consists of one of the most important etiologies that leads to the presence of intracranial stenosis [59,60]. According to several studies, the OR fluctuates among 1.9 (Bae et al.), 4 (Uehara et al.) and 5.9 (Huang et al.) for those populations [1,61,62,63]. Since its appearance is more frequent among people from Asia, several observational studies have been conducted on data from those populations. Thomas et al. have gathered information of a total 18,279 patients for 8.32 years. The results have shown that among 191 identified deaths, the presence of middle cerebral artery (MCA) stenosis is an independent predictor of higher vascular mortality in subjects with T2DM [64]. Lastly, another interesting survey about intracranial stenosis by Thomas et al. showed that among patients with T2DM, albuminuria and hypertension are connected to asymptomatic MCA stenosis [65]. In addition, the results of this study indicate that MCA stenosis is linked to higher vascular mortality rates [65].

Ferris et al. [66] investigated whether DM can affect structural and metabolic characteristics of cerebral tissue in individuals with stroke. DM subjects had lower cortical thickness and creatine bilaterally in the sensorimotor cortex [66]. Regional cortical thickness in the primary and secondary sensorimotor cortices was reduced bilaterally, whereas the volume of cortical grey matter or cerebral white matter did not differ between DM and non-DM subjects [66]. These results are supported by several other studies [67,68,69]. Increased inflammatory factors and reduced cortical plasticity are the major underlying mechanisms proposed for these differences [59,60,61,62,63]. Moreover, the reduced sensorimotor thickness may, perhaps, explain the more inadequate post-stroke recovery of DM subjects [66].

In practice, the lacunar infarct is still the commonest stroke type in DM [70]. This has been attributed to microvascular disease and concurrent hypertension [70].

## 4. Pathophysiology

**Figure 1:**
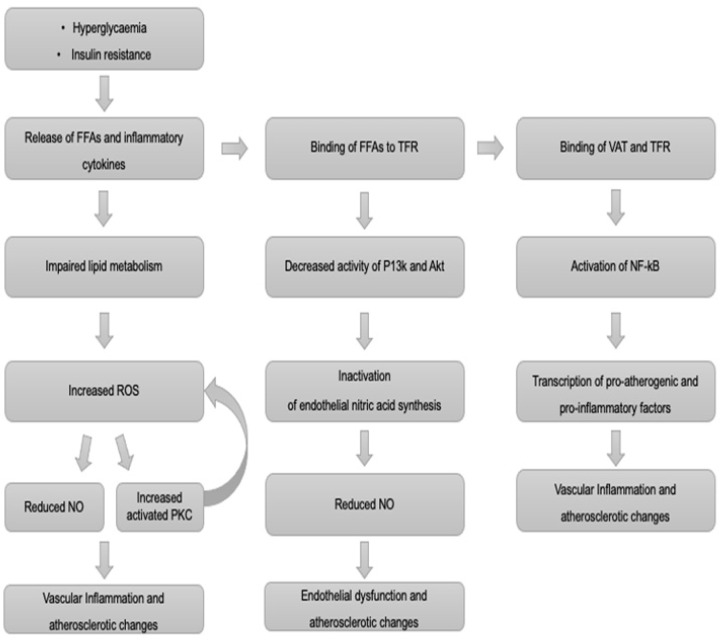
Main pathways that result in vascular inflammation, formation of atherosclerotic changes and endothelial dysfunction, culminating in increased stroke risk.

**Figure 2:**
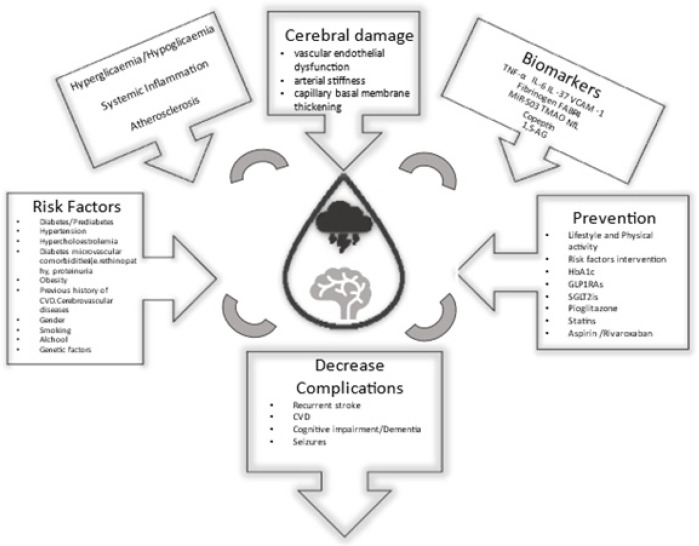
Stroke and diabetes risk factors, pathophysiology, complications and prevention for stroke promoted by diabetes.

To date, several pathophysiological mechanisms have been associated with stroke in DM, including vascular endothelial dysfunction, increased early age arterial stiffness, systemic inflammation and thickening of the basal capillary membrane [20,71,72]. Taken together, these pathologies may be seen as a form of premature vascular aging [20,71,72]. Their main mechanisms are summarised in Figure 1 and Figure 2.

The role of systemic inflammation in the development of atherosclerotic plaques is important. Various indicators have been associated with severe systemic inflammation and CVD, notably reduced adiponectin [9].

Hyperglycaemia and insulin resistance represent independent factors contributing to the development of atherosclerosis [9,72]. Adipose tissue releases free fatty acids (FFAs) and inflammatory cytokines [9,71,73,74]. The latter impair lipid metabolism, leading to an increased production of reactive oxygen species (ROS) and, ultimately, increasing systemic inflammation [9,65,67,68]. When FFAs bind to their receptor (TFR), the activity of P13 kinase (P13k) decreases. As a result, endothelial nitric acid synthesis and nitric oxide (NO) production are reduced [9,71,73,74].

NO is a key molecule for maintaining normal endothelial cell function, and thus its reduction causes endothelial dysfunction and atherosclerotic changes [71,73,74].

Increased ROS due to long-standing hyperglycaemia cause transcription and expression of genes encoding inflammatory factors through the upregulation of NF-κΒ. They also upregulate protein kinase C (PKC) production [71,75,76].

Protein kinase C (PKC) upregulates the production of endothelin-1 (ET-1), which is involved in platelet aggregation and vasoconstriction [65,69,70]. PKC also increases the expression of cyclo-oxygenase 2 (COX-2), thereby increasing thromboxane A2 (TXA2) and decreasing prostacyclin (PGI2) [71,75,76]. Of note, PKC further increases ROS production and NO reduction, creating a vicious circle [71,72,73,74,75,76,77,78]. These perturbations favour the progression of atherosclerosis [9,71,76].

In addition, binding of visceral adipose tissue to TFR activates the nuclear factor NF-kB, which, in turn, promotes the transcription of pro-atherogenic and pro-inflammatory factors [68,70,71,72]. Under normal circumstances, insulin inhibits thrombosis and increases fibrinolysis. By contrast, insulin resistance leads to a pro-thrombotic state. This is reflected in an increased calcium concentration in platelets, leading to their aggregation [9,71,73,74,75,76].

Moreover, long-term hyperglycaemia activates the polyol pathway [71,73]. It also increases the formation of advanced glycation end products (AGEs), as well as the expression of their receptor (RAGE) and its activating ligands [73]. These pathways aggravate and perpetuate atherosclerosis [71,73,76].

Recurrent hypoglycaemia may also be considered a risk factor for stroke, inasmuch as it could promote a thrombotic state. A recent study has established that the relative risk for stroke was 1.75 among T2DM subjects receiving sulphonylureas and sustaining >3 hypoglycaemic events [79]. Further investigations are need on this possible correlation [80].

## 5. Management of Hyperglycaemia during Acute Stroke

Proper glucose management in patients with acute stroke plays a crucial role in the course of the incident. Therefore, both hyperglycaemia and hypoglycaemia should be monitored and avoided in the acute phase. The American Heart Association (AHA)/American Stroke Association guidelines suggest close monitoring to prevent hyperglycaemia, while the recommended levels of blood glucose are between 140–180 mg/dL (7.8–10 mmol/L) during the first 24 h. The European Stroke Initiative guidelines indicate immediate management of blood glucose of 180 mg/dL (10 mmol/L) or higher [78,81].

Studies have shown that impaired blood glucose levels can result in unfavourable results in both ischaemic and haemorrhagic stroke since it increases brain lactate production and reduces salvage of penumbral tissue, therefore, leading to a greater infarct size. Moreover, the increase in oxidative stress and systematic inflammation induces worse stroke consequences [82].

In addition, stress hyperglycemia has been reported as an independent predictor of stroke recurrence but has also been associated with a negative ischemic outcome [82,83]. Stress hyperglycemia is referred to as hyperglycemia resulting from an acute stress situation, such as a stroke [82,83]. Studies have shown that a large number of patients with stroke have high levels of hyperglycemia during their hospitalization [83,84]. A study by Pan et al. showed that patients with stress hyperglycemia were at a high risk of recurrence of a stroke within 90 days of onset. Specifically, 3044 patients participated in the study, of which 9.9% had a second episode within 90 days, 48% of whom had hyperglycemic stress [85]. Another large study of 8622 participants with ischemic stroke showed that hyperglycemic stress, measured using the Glucose/HbA1c ratio, is associated with a severe neurological deficit over a one-year period [9].

Generally, subcutaneous insulin is used to manage hyperglycaemia in acute stroke. According to GRASP (Glucose regulation in acute stroke patients) and THIS (Treatment of hyperglycemia in ischemic stroke) trials, intensive glucose control through the use of insulin in patients with acute ischaemic stroke is both safe and results in better control [86,87]. However, there are also studies suggesting that intensive insulin therapy has no difference compared to usual care and appears to have a higher rate of hypoglycaemic episodes. Hypoglycaemia should be also avoided, since it is linked to an increased risk of mortality [88].

A large randomized clinical trial, the SHINE trial, examined the contribution of intensive treatment of hyperglycaemia during acute ischemic stroke. For this purpose, 1151 patients participated in this study and received either continuous intravenous insulin or insulin on a sliding scale. The results did not demonstrate a remarkable difference between the two groups; therefore, they supported the conclusion that intensive glucose control is not vital [89].

## 6. Prevention

Primary prevention of stroke in DM addresses lifestyle and other risk factors. Of note, during the last few years, a protective role in terms of CVD has been discussed for anti-diabetic treatment as well [90,91]. In terms of stroke, the most important new data have been obtained with glucagon-like peptide 1 receptor agonists (GLP-1RAs) [91] (Table 1).

The REWIND (Researching Cardiovascular Events With a Weekly Incretin in Diabetes) RCT [92] was a large multi-centre randomised controlled trial (RCT) comparing dulaglutide with a placebo. It showed an overall reduction in stroke (HR: 0.76, 95% CI: 0.62–0.94, *p* = 0.010), reduction in ischaemic stroke (HR: 0.75, 95% CI: 0.59–0.94, *p* = 0.012), reduction in disabling stroke (HR: 0.74, 95% CI: 0.56–0.99, *p* = 0.042) and reduction in the composite outcome of nonfatal stroke or death (HR: 0.88, 95% CI: 0.79–0.98, *p* = 0.017), but not on haemorrhagic stroke or stroke severity [92,93].

The ELIXA (Evaluation of Lixisenatide in Acute Coronary Syndrome) RCT [75] compared lixisenatide with a placebo in terms of CVD. There was no effect on stroke (HR: 1.12, 95% CI: 0.79–1.58) [94].

The LEADER (Liraglutide Effect and Action in Diabetes: Evaluation of Cardiovascular Outcome Results) RCT [95] studied the cardiovascular effects of liraglutide. This agent significantly reduced cardiovascular (HR: 0.78, 95% CI: 0.66–0.93, *p* = 0.007) and total (HR: 0.85, 95% CI: 0.74–0.97, *p* = 0.02) mortality, while it insignificantly reduced stroke (HR: 0.86, 95% CI: 0.71–1.06), non-fatal stroke (HR: 0.86, 95% CI: 0.72–1.11) and fatal stroke (HR: 0.64, 95% CI: 0.34–1.19) [95].

The SUSTAIN (Semaglutide and Cardiovascular Outcomes in Patients with Type 2 Diabetes)-6 trial [96] examined the cardiovascular effects of semaglutide. This agent reduced non-fatal stroke by 39% (HR: 0.61, 95% CI: 0.38–0.99, *p* = 0.04) and non-fatal MI by 26% (HR: 0.74, 95% CI: 0.51–1.08, *p* = 0.12) [96].

The EXCEL (Exenatide Study of Cardiovascular Event Lowering Trial) trial [97] compared exenatide with a placebo. The incidence of major adverse cardiovascular events did not differ between the two groups [97]. The HARMONY (Albiglutide and cardiovascular outcomes in patients with type 2 diabetes and cardiovascular disease) trial [92] compared albiglutide with a placebo. Albiglutide was superior to the placebo (*p* < 0.0001 for non-inferiority, *p* = 0.0006 for superiority) in reducing overall major cardiovascular events (HR: 0.78, 95% CI: 0.68–0.90), but not stroke in particular [98]. The PIONEER (Oral Semaglutide and Cardiovascular Outcomes in Patients with Type 2 Diabetes) trial [99] compared semaglutide with a placebo and demonstrated the cardiovascular safety of semaglutide. Indeed, this agent was not inferior to the placebo and numerically reduced non-fatal stroke (HR: 0.74; 95% CI: 0.35–1.57) [99].

Overall, GLP-1RAs have demonstrated beneficial actions in stroke, as confirmed by large systematic reviews and meta-analyses [100,101]. This holds mainly true for dulaglutide [92] and semaglutide [96], secondarily also for liraglutide [95] and oral semaglutide [99].

Another important new class of oral anti-diabetic agents is sodium-glucose cotransporter-2 inhibitors (SGLT-2is) [102]. These have achieved impressive results in terms of reducing heart failure and renal outcomes [102] and, according to international guidelines [103], should be used in subjects with established CVD, irrespective of their glycaemic control. In subjects with established CVD, they also reduce the composite endpoint including cardiovascular death, non-fatal MI and non-fatal stroke [102]. However, they have not been shown to specifically reduce stroke per se [102]. Indeed, a very recent national Swedish registry has concluded that GLP-1RAs mainly reduced stroke and peripheral artery disease, whereas SGLT-2is mainly reduced heart failure and total mortality [104].

The ROCKET AF (Rivaroxaban Once-daily, Oral, Direct Factor Xa Inhibition Compared with Vitamin K Antagonism for Prevention of Stroke and Embolism Trial in Atrial Fibrillation) trial [105] examined the efficacy and safety of rivaroxaban vs. warfarin in DM subjects with atrial fibrillation (AF). The efficacy and safety of rivaroxaban compared with warfarin were similar in patients with and without DM, supporting the use of rivaroxaban as an alternative to warfarin to prevent stroke in patients with DM and AF [105]. A subsequent meta-analysis has demonstrated the equal efficacy and safety of novel oral anticoagulants and warfarin in DM subjects with AF [106].

Physical activity and weight reduction are advisable for the secondary prevention of stroke and MI [107,108]. While their effects have been more extensively studied in MI, these measures favourably impact on overall cardiovascular benefit [107,108,109,110]. For this purpose, antidiabetic agents promoting weight loss or being weight-neutral are, generally, preferable [111]. In more severe obesity (BMI > 40 kg/m^2^), metabolic surgery may also be considered [112]. Finally, magnesium-rich diets have been shown to provide some additional protection against stroke in DM [113].

High HbA_1c_ has been associated with an increased risk of first-ever stroke in patients with DM [114]. Thus, adequate glycaemic control is a foremost priority. Nevertheless, the contribution of intensive glycaemic control to stroke reduction remains controversial. The Diabetes Control and Complications Trial/Epidemiology of Diabetes Interventions and Complications (DCCT/EDIC) study has shown that intensive glycemic control reduces CVD in T1DM [115]. However, data from other studies demonstrate that intensive glycaemic control does not reduce stroke incidence [116,117]. In the Steno-2 trial, intensive glycaemic control achieved a beneficial effect on cardiovascular events when combined with multi-factorial risk management [118].

Blood pressure reduction is of paramount importance for the prevention of stroke in DM [119]. Most guidelines recommend a blood pressure target of <140/90 mm Hg [120]. However, a different target applies to young individuals with DM and those with DM and microalbuminuria: ≤130/80 mmHg. In the United Kingdom Prospective Diabetes Study (UKPDS), T2DM subjects with lower blood pressure exhibited a 44% reduction in stroke incidence than the group with less stringent blood pressure control [120]. Similarly, data from the Action to Control Cardiovascular Risk in Diabetes (ACCORD) and the ACCORD Blood Pressure BP trials indicate that intensive systolic blood pressure control is linked with a lower risk of stroke [43]. However, systolic blood < 120 mmHg increased severe adverse effects and, therefore, should be avoided [121].

As shown by the Heart Protection Study and Collaborative Atorvastatin Diabetes Study, statins are valuable in lowering LDL and thus improving cardiovascular risk [122,123]. In addition, a recent study has shown that a combination of statin and ezetimibe results in further LDL reduction and could benefit people with DM [124].

Aspirin is recommended as an initial agent for the secondary prevention in patients with T2DM that have experienced a stroke. In this setting, clopidogrel has been shown to be non-inferior [125].

Finally, pioglitazone should be considered as a therapeutic option for patients with DM or insulin resistance as a factor of secondary prevention [126,127,128,129]. After a Stroke (IRIS) trial, the Insulin Resistance Intervention demonstrated a reduction in non-fatal stroke with pioglitazone compared with a placebo [119]. The significant reduction in stroke risk (HR: 0.68, 95% CI: 0.50–0.92, *p* = 0.01) has also been confirmed in a meta-analysis [129].

Regarding specialized treatments, such as thrombectomy and thrombolysis, the majority presented data are unfavorable for people with DM. In a study by Borggrefe et al., patients with stroke in either the terminal internal carotid artery and/or middle cerebral artery participated. After comparing diabetic and non-diabetic patients who underwent thrombectomy, the data showed poorer outcomes for the patients with DM based on modified Rankin Scale after 90 days (mRS 90) (mRS90 > 2, *p* < 0.05), while the outcomes were associated with poor glycemic control and older age [130]. The hypothesis that poor glycemic control has a negative effect on thrombectomy results is also supported by other studies. Chang et al. made a comparative study in diabetic and non-diabetic patients with stroke located in large vessels and showed that elevated levels of HbA1c upon admission are an independent factor that hinders good functional recovery after treatment [131].

In terms of thrombolytic therapy, the presented data are similar. In the literature, there is a large study showing that poor glycemic regulation is a poor prognostic factor for recovery after thrombosis, and in particular, high intake blood glucose levels [132]. However, these data are not exclusively associated with patients with DM, as they also relate to stress hyperglycemia. A big meta-analysis, however, suggests that diabetes itself may be a poor prognostic factor for this kind of treatment, as it has been associated with adverse treatment-related outcomes (OR, 0.77; 95% CI, 0.69–0.87) [133].

## 7. Emerging Biomarkers

It is being increasingly appreciated that we need to identify biomarkers of stroke. These should help us predict and evaluate the risk of stroke in DM. To date, several biomarkers have been examined for this purpose. However, none of them have been unequivocally confirmed as reliable and useful.

AGEs are formed by protein glycation during long-standing hyperglycaemia. They bind to their membrane receptor (RAGE) and alter signalling and gene expression [134]. This reaction promotes inflammation and oxidative stress through an increased production and secretion of pro-inflammatory cytokines [135]. It has been suggested that AGEs could participate in the development of macrovascular complication, while soluble forms of RAGE could play a key role in the protection from atherosclerosis in T1DM [136].

Several inflammatory cytokines have been discussed [137,138,139]. Tumour necrosis factor-alpha (TNF-α) appears to promote atherosclerosis. The same holds true for interleukin-6 (IL-6) and interleukin-37 (IL-37), whose low levels may have a cardioprotective role [138,139].

Vascular cell adhesion molecule 1 (VCAM-1) plays a key role in angiogenesis. A positive correlation of VCAM-1 with both microvascular and macrovascular DM complications, including stroke, has been suggested, but not yet unequivocally demonstrated [140,141].

Perfluoroalkyl substances (perfluorohexane sulphate, C8-perfluorooctanoic acid, perfluoroctane sulfonate and perfluorononaoic acid) have also been examined [136]. Interestingly, an adverse correlation of perfluorohexane sulphate and perfluoroctane sulfonate with stroke in DM has been reported [142].

Lee et al. [143] showed that increased serum fibrinogen is associated with early neurological deterioration in DM subjects with stroke. Moreover, micro-ribonucleic acid 503 (MiR-503) has been examined as a marker of ischaemic stroke and its severity in DM [144].

Adipocyte fatty acid-binding protein 4 (FABP-4), which is expressed in adipose tissue and may promote atherosclerosis, has been examined as a biomarker of stroke and MI [145]. Regarding the latter, results are disappointing, but in regard to the former, it appears that further enquiry is warranted [145].

Trimethylamine N-Oxide (TMAO) is a molecule generated via gut microbial metabolism and is affected by diet, gut microbia and drug use [146,147]. The accumulating evidence suggests that an elevated TMAO may indicate an increased CVD risk, including stroke, especially in T1DM [148].

A 7-year prospective study [149] suggested that the neurofilament light chain (NfL) level may indicate brain injury and risk of stroke. Indeed, a follow-up showed that NfL was associated with subclinical ischaemic vascular disease, which increased the risk of ischaemic stroke [149]. This was described to brain cellular death from cerebral ischaemia, which released brain structural proteins (including NFL) through the blood–brain barrier in circulation [150].

Another potential biomarker is copeptin. This appears to be a predictor of stroke, MI and coronary artery disease, especially in DM [150,151,152].

Lastly, low serum 1,5-anhydroglucitol (1,5-AG) levels have been shown to be a potential risk marker of stroke well-controlled DM [153].

## 8. Conclusions

Diabetes remains one of the leading risk factors for stroke: subjects with diabetes carry a two- to tenfold risk of stroke (Table 2) [5]. Various factors are implicated in this risk. These are related to lifestyle, comorbidities, hypertension and lipid profile, but also anthropometric data, such as body weight [12,18,19,20,21,22,23,24,25,26,27,28,29,30,31,32,33,34,35,36,37,38,39,40,41,42,43,44,45,51] (Figure 2).

Numerous underlying pathophysiological mechanisms involved have now begun to be clarified. These include insulin resistance and a chronic hyperglycaemic environment that leads to impaired adipose tissue homeostasis. Through various pathways, these perturbations lead to systemic inflammation, vascular dysfunction and atherosclerosis. Taken together, these pathologies may be seen as a form of premature vascular aging, ultimately predisposing to stroke [9,71,72,73,74,75,76,77,78].

Prevention of cardiovascular complications remains one of the most important goals for people with diabetes. Hence, it is recommended to address hyperglycaemia and other vascular co-morbidities, as well as body weight [43,105,106,107,108,109,110,111,112,113,114,115,116,117,118,119,120,121]. In recent years, GLP1-RAs and pioglitazone have become very promising in stroke prevention [91,92,93,94,95,96,97,128,129].

Finally, biomarkers have been sought to improve stroke prognosis and assessment. Despite their plethora, no convincing findings have yet established their use [134,135,136,137,138,139,140,141,142,143,144,145,146,147,148,149,150,151,152,153].

## Figures and Tables

**Table 1:** Effects of glucagon-like peptide-1 receptor agonists (GLP-1RAs) and sodium-glucose transporter inhibitors (SGLT-2is) on risk of fatal and non-fatal stroke in randomized controlled trials in patients with type 2 diabetes mellitus. Results are expressed as hazard ratio compared to placebo.

Trial	Duration	Participants	Drug	Dose	HR (95% Cl)	*p* Value
REWIND	2 years	9901	Dulaglutide	1.5 mg/week	0.76	0.01
ELIXA	2.1 years	6068	Lixisenatide	20 μg/day	1.12	0.54
LEADER	3.8 years	9340	Liraglutide	1.8 mg/day	0.86	0.16
SUSTAIN 6	2.1 years	3297	Semaglutide	0.5 mg or 1 mg/week	0.65	0.06
HARMONY	1.5 years	9463	Albiglutide	30 mg or 50 mg/week	0.86	0.30
EMPA-REG	3.1 years	7020	Empagliflozin	10 mg or 25 mg/day	1.18	0.26
CANVAS	188.2 weeks	10,142	Canagliflozin	100 mg or 300 mg/day	0.87	0.02

**Table 2:** Article highlights (according to cited references).

Article Highlights
Epidemiology and Risk Factors People with DM have with a two- to tenfold risk of stroke [5,9,10] Pre-diabetes is also linked with an increased risk of stroke [13] Comorbidities, such as hypertension, dyslipidaemia, previous MI and heart failure, increase the risk of stroke [31,32,33,34] Microvascular diabetic complications are associated with an increased risk of stroke, as well [43,44]
Clinical manifestations Ischaemic and haemmorrhagic stroke [45] DM is associated with commoner posterior circulation brain infarction and brain stem infarction [46] DM subjects had lower cortical thickness and creatine bilaterally in the sensorimotor cortex [47]
Pathophysiology Systemic inflammation is important in the development of atherosclerotic plaques [9] Reduced endothelial NO production causes endothelial dysfunction and atherosclerotic changes [4,55,56] Activation of nuclear factor NF-kB promotes the transcription of pro-atherogenic and pro-inflammatory factors [53,55,56] Hyperglycaemia activates the polyol pathway, increases formation of AGEs and RAGE, thus promoting atherosclerosis [53,55]
Management of hyperglycaemia during acute stroke Suggested levels of blood glucose are between 140–180mg/dl (7.8–10 mmol/L) [59] Intensive glucose control in the acute phase is controversial [61,62,63,64,65]
Prevention Some GLP-1RAs, including dulaglutide, liraglutide and semaglutide, offer protection against stroke according to cardioprotection studies [66,67,68,69,70,71,72,73,74,75] Rivaroxaban can be used as an alternative to warfarin for stroke prevention [82] Physical activity and weight reduction are advisable for secondary prevention [83,84] Meticulous management of hyperglycaemia and hypertension can reduce stroke incidence [89,90,91,92,93,94,95,96,97,98]
Emerging Biomarkers Several biomarkers have been examined for the prediction of stroke, but none of them have been unequivocally confirmed as reliable and useful [43,107,108,109,110,111,112,113,114,115,116,117,118,119,120,121,122,123,124]

## Data Availability

Not applicable.

## Abbreviations

DM	Diabetes Mellitus
T2DM	type 2 DM
T1DM	type 1 DM
MI	myocardial infarction
BMI	body-mass index
HRs	hazard ratios
CI	confidence Interval
MCA	middle cerebral artery
FFAs	free fatty acids
ROS	reactive oxygen species
TFR	Transferrin receptor
P13k	P13 kinase
NO	nitric oxide
NF-κΒ	nuclear factor kappa-light-chain-enhancer of activated B cells
PKC	protein kinase C
ET-1	endothelin-1
COX-2	cyclo-oxygenase 2
TXA2	thromboxane A2
PGI2	prostacyclin
AGEs	advanced glycation end products
RAGE	Receptor for advanced glycation endproducts
AHA	American Heart Association
GLP-1RAs	glucagon-like peptide 1 receptor agonists
REWIND	Researching Cardiovascular Events With a Weekly Incretin in Diabetes trial
ELIXA	Evaluation of Lixisenatide in Acute Coronary Syndrome trial
LEADER	Liraglutide Effect and Action in Diabetes: Evaluation of Cardiovascular Outcome Results
SUSTAIN	Semaglutide and Cardiovascular Outcomes in Patients with Type 2 Diabetes
EXCEL	Exenatide Study of Cardiovascular Event Lowering Trial
HARMONY	Albiglutide and cardiovascular outcomes in patients with type 2 diabetes and cardiovascular disease
PIONEER	Oral Semaglutide and Cardiovascular Outcomes in Patients with Type 2 Diabetes trial
SGLT-2is	sodium-glucose cotransporter-2 inhibitors
ROCKET AF	(Rivaroxaban Once-daily, Oral, Direct Factor Xa Inhibition Compared with Vitamin K Antagonism for Prevention of Stroke and Embolism Trial in Atrial Fibrillation trial
DCCT/EDIC	Diabetes Control and Complications Trial/Epidemiology of Diabetes Interventions and Complications
UKPDS	United Kingdom Prospective Diabetes Study
BP	blood pressure
HbA_1c_	Glycated haemoglobin
ACCORD	Action to Control Cardiovascular Risk in Diabetes
TNF-α	Tumour necrosis factor-alpha
IL-6	Interleukin-6
IL-37	interleukin-37
VCAM-1	Vascular cell adhesion molecule 1
MiR-503	micro-ribonucleic acid 503
FABP-4	Adipocyte fatty acid-binding protein 4
TMAO	Trimethylamine N-Oxide
NfL	neurofilament light chain
1,5-AG	1,5-anhydroglucitol
